# Dental rolls for tendon repair practice

**DOI:** 10.1308/003588413X13511609958055b

**Published:** 2013-03

**Authors:** L Li, A McKee

**Affiliations:** Peterborough and Stamford Hospitals NHS Foundation Trust, UK

**Figure 1:**
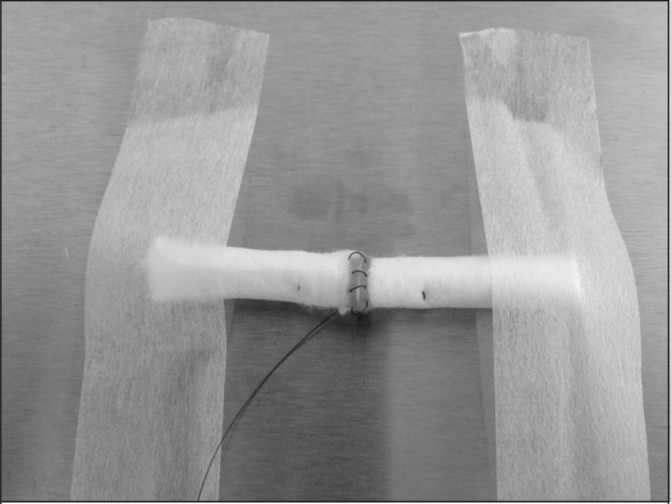
Core suture placed 10mm from inside edge; epitendon suture placed 2mm from inside edge

An inexpensive and readily available material for practising tendon repair sutures is dental rolls. Two dental rolls secured to a table and placed longitudinally opposite each other can simulate the cut ends of a tendon (Fig 1). The size and shape of the dental roll is an acceptable substitute for tendon, and the feel of the bite is also comparable. This technique allows placement of the posterior wall epitendon suture first and permits testing of the construct mechanics at each stage. This is a useful technique and a valuable adjunct to any surgical skills course. It also avoids the need to dispose of animal materials.

